# Ready for goal setting? Process evaluation of a patient-specific goal-setting method in physiotherapy

**DOI:** 10.1186/s12913-017-2557-9

**Published:** 2017-08-31

**Authors:** Anita Stevens, Albère Köke, Trudy van der Weijden, Anna Beurskens

**Affiliations:** 1 Zuyd University of Applied Sciences, Faculty of Health, P.O. Box 550, 6400 AN Heerlen, the Netherlands; 2 Department of Family Medicine, Maastricht University, CAPHRI Care and Public Health Research Institute, Maastricht, the Netherlands; 3 Adelante Centre of Research in Rehabilitation, Hoensbroek, the Netherlands; 4 Department of Rehabilitation Medicine, Maastricht University, Maastricht, the Netherlands

## Abstract

**Background:**

Patient participation and goal setting appear to be difficult in daily physiotherapy practice, and practical methods are lacking. An existing patient-specific instrument, Patient-Specific Complaints (PSC), was therefore optimized into a new Patient Specific Goal-setting method (PSG). The aims of this study were to examine the feasibility of the PSG in daily physiotherapy practice, and to explore the potential impact of the new method.

**Methods:**

We conducted a process evaluation within a non-controlled intervention study. Community-based physiotherapists were instructed on how to work with the PSG in three group training sessions. The PSG is a six-step method embedded across the physiotherapy process, in which patients are stimulated to participate in the goal-setting process by: identifying problematic activities, prioritizing them, scoring their abilities, setting goals, planning and evaluating. Quantitative and qualitative data were collected among patients and physiotherapists by recording consultations and assessing patient files, questionnaires and written reflection reports.

**Results:**

Data were collected from 51 physiotherapists and 218 patients, and 38 recordings and 219 patient files were analysed. The PSG steps were performed as intended, but the ‘setting goals’ and ‘planning treatment’ steps were not performed in detail. The patients and physiotherapists were positive about the method, and the physiotherapists perceived increased patient participation. They became aware of the importance of engaging patients in a dialogue, instead of focusing on gathering information. The lack of integration in the electronic patient system was a major barrier for optimal use in practice. Although the self-reported actual use of the PSG, i.e. informing and involving patients, and client-centred competences had improved, this was not completely confirmed by the objectively observed behaviour.

**Conclusion:**

The PSG is a feasible method and tends to have impact on increasing patient participation in the goal-setting process. However, its full potential for shared goal setting has not been utilized yet. More implementation effort is needed to achieve the required behaviour change and a truly client-centred attitude, to make physiotherapists totally ready for shared goal setting.

**Electronic supplementary material:**

The online version of this article (doi:10.1186/s12913-017-2557-9) contains supplementary material, which is available to authorized users.

## Background

Healthcare is currently developing from a disease-outcome paradigm towards a goal-oriented paradigm [1]. This implies an increased focus on the patient’s individual goals, and an active role of patients in articulating their preferences and needs. Physiotherapists have to find ways to actively involve patients in goal setting and treatment planning, thus delivering client-centred care [2, 3]. Patient participation is considered an important element in optimizing treatment adherence, motivation and patient satisfaction with therapy [4, 5]. In daily practice, however, patient participation appears to be challenging [6–13], and several studies have shown that patients are hardly involved in goal setting [6–10, 14, 15]. Professional guidelines emphasize the importance of shared goal setting, and practical tools are needed to support this process. A structured approach to goal setting can have a positive effect on patient participation, client-centeredness and efficiency [5, 15–20]. The use of patient-specific measurement instruments can support the process of goal setting in different phases [19]. One such instrument is the Patient-Specific Complaints (PSC) instrument, [21] which is similar to the Patient-Specific Functional Scale (PSFS) [22]. The original PSC involves four steps, in which the patient’s main activity problems are identified, prioritized, scored and evaluated. It is a frequently used instrument in Dutch physiotherapy practices [23]. Recent analysis showed, however, that its potential for goal setting was not fully utilized [6, 8]. Hence, the existing PSC instrument was optimized in collaboration with patients and physiotherapists. Four main objectives for improvement were processed into the original PSC: 1) a clear administration procedure; 2) targeted use within the physiotherapy process; 3) using client-activating communication skills; and 4) a client-centred attitude of the physiotherapist. The PSC instrument was further developed into a six-step method, the so-called PSG, and was integrated across the entire physiotherapy process [24]. The PSG offers physiotherapists a structured method to involve patients in the goal-setting process, from problem identification, setting goals, and treatment planning up to evaluation. The method is based on a goal-setting framework [20] and shared decision making [25].

The first aim of this study was to examine the feasibility of the PSG in daily physiotherapy practice. Therefore, a process evaluation was conducted, which is recommended prior to studying the effect and large-scale implementation of a new method [26]. Therefore we wanted to get insight into the extent to which all components of the PSG were actually used as intended, to examine the views of its users, and to determine the influence of factors that may affect its effective implementation [26, 27]. The following research questions were formulated: 1a. To what extent is the PSG performed as intended? 1b. How satisfied are patients about the PSG? 1c. What are the physiotherapists’ experiences with the PSG?

The second aim of this study was to explore the potential impact of the new method. Therefore, the following research question was formulated: 2. Did the physiotherapists’ intention to use the PSG, the actual use of the PSG, and their client-centred competences improve?

## Methods

### Design

A process evaluation, using the elements of Saunders et al. [27] in a non-controlled intervention study. Quantitative and qualitative data were used to examine the feasibility and the potential impact of the PSG. The study was conducted between September 2015 and March 2016.

### Setting and participants

The study was carried out in private physiotherapy practices in community-based healthcare in the southern and north-western region of the Netherlands. The practices were purposively sampled by the first author from the database of internship supervisors from the Bachelor physiotherapy at Zuyd University of applied sciences Heerlen, and a physiotherapy network Noord-Holland, according the following criteria: treating adult patients, preferably chronic patients, and using the (original) PSC. The physiotherapists were invited to participate by means of an invitation letter with information about the study and the training, and were contacted by phone after 1 week. The physiotherapists approached their (preferably chronic) patients for participation, i.e., asked whether they would be willing to have a consultation recorded and fill in a questionnaire.

### Ethical considerations

Patients and physiotherapists were informed by means a written information letter, and written informed consent was obtained before the recordings. All data were given an identification number and were anonymously processed.

### Intervention

The intervention comprised the application of the PSG in physiotherapy including a training course. The PSG is a method to support physiotherapy goal setting and consists of six steps that are integrated in the physiotherapy process [24]: 1) identifying problematic activities the patient encounters in daily life as a consequence of their health problem; 2) prioritizing the most important activity he/she wants to work on; 3) scoring the perceived ability to perform the selected activity on an 11-point Numeric Rating Scale (0 = impossible to perform, 10 = easy to perform) (in the original PSC instrument, a higher score meant poorer performance, but this was reversed in the new PSG method); 4) setting goals, i.e. translating the selected activities into treatment goals; 5) planning treatment, i.e. making a shared decision about the treatment plan; and 6) evaluating the treatment goals. Essential elements to stimulate patient participation are informing the patient by introducing every step and explaining its rationale, and the use of client-activating communication skills.

The physiotherapists were trained in three 3-h training sessions, showing them how to use the PSG in practice [24]. The training course was accredited with 15 quality credit points awarded by the Royal Dutch Society for Physiotherapy (Koninklijk Nederlands Genootschap Fysiotherapie, KNGF). The learning aims were based on the four main objectives for improvement of the PSC instrument, and formulated as follows: the physiotherapist is: 1) aware of the content and rationale of the PSG and is able to apply the instrument correctly; 2) able to fully integrate the PSG across the physiotherapy process; 3) able to communicate with patients in a client-centred manner, within the process of goal setting; and 4) is aware of what is meant by client-centeredness, and is able to provide client-centred physiotherapy using the PSG. An instruction manual supported the physiotherapists during the training course. All training sessions were interactive and practical, including role plays (with simulated patients), video observations and peer assessment with feedback on video recordings.

### Variables and data collection

Data for the process evaluation, i.e., the feasibility of the PSG, were collected during the six-week follow-up period after the last training session, by means of structured observation of video/audio tapes and patient files, questionnaires and reflection reports (Table 1). The key elements of process evaluation [27] were operationalized after which suitable assessments were developed. The assessments had all been pre-tested and evaluated in a previous field test [24].

**Table 1:** Research questions, timing of data collection and measurements used

	Physiotherapist					Patient
Timing of data collection	Before training	At follow-up				
Measurements	Questionnaire	Questionnaire	Observation video/audio	Assessing patient files	Reflection report	Questionnaire
1a. To what extent is the PSG performed as intended?						
Performance and recording of the PSG steps			X	X		
Patients’ involvement in the PSG						X
1b. How satisfied are patients about the PSG?						
Patients’ satisfaction with the PSG						X
1c. What are physiotherapists’ experiences with the PSG?						
Physiotherapists’ experiences with the PSG					X	
2. Did the physiotherapists’ intention to use the PSG, the actual use of the PSG, and their client-centred competences improve after training?						
Intention to use the PSG	X	X				
Actual use of the PSG	X	X				
Client-centred competences	X	X				

For research question 1a: *To what extent is the PSG performed as intended?*, the element ‘dose delivered’, i.e. the completeness of the PSG, was operationalized by the performance and recording of the PSG steps (Table 2). The element ‘fidelity’, or integrity, i.e. the extent to which the intervention was applied in a manner consistent with the instructions, was operationalized by the performance and recording of the detailed components of each step (Table 2). Data were collected by the physiotherapists, who video- or audiotaped one initial consultation of a patient using the PSG. They also handed in five anonymized copies of electronic patient files from patients who had had a new encounter during the follow-up period. The ‘dose received exposure’, i.e. the extent of involvement of participants in the intervention, was operationalized as the patients’ involvement in the PSG. Therefore, patients filled in a 4-item questionnaire about their awareness of their treatment goals and treatment plan (Table 3).

**Table 2:** Observed and recorded performance of the PSG

Observed performance in the video/audio tapes *N* = 38^a^		Present
PSG Steps	Components per step	*n* (%)
Step 1 Identification	(*n* = 29)	29 (100)
	Introduces ‘identification’ step in advance	24 (83)
	Explains reason for identification	13 (45)
	Stimulates patient to think about problematic activities	28 (97)
	Specifies the activities in concrete terms	29 (100)
	Summarizes the activities identified	24 (83)
Step 2 Prioritization	(*n* = 30)	28 (93)
Step 3 Scoring	(*n* = 32)	32 (100)
	Introduces ‘scoring’ step in advance	26 (81)
	Explains reason for scoring	9 (28)
	Scores correctly (positive)	29 (91)
Step 4 Setting goals	(*n* = 35)	35 (100)
	Introduces ‘setting goals’ step in advance	23 (66)
	Explains reason for setting goals	10 (29)
	Repeats selected problematic activities	25 (71)
	Discusses physical examination results with patient	19 (54)
	Relates physical examination results to selected problematic activities	10 (29)
	Asks patient about treatment goals	28 (80)
	Summarizes the treatment goals discussed	18 (51)
	Checks patient’s understanding	10 (29)
Step 5 Planning treatment	(*n* = 33)	32 (97)
	Introduces ‘planning treatment’ step in advance	9 (27)
	Explains reason for planning treatment	4 (12)
	Informs patient about treatment options	27 (82)
	Elicits patient’s preferences for treatment	20 (61)
	Incorporates patient’s preferences in treatment plan	14 (42)
	Summarizes the treatment plan	12 (36)
	Checks patient’s understanding	7 (21)
Recorded performance in the patient files *N* = 218		Present
PSG steps	Components per step	n (%)
Step 1 Identification		216 (99)
Step 4 Setting goals		215 (99)
	Selected problematic activities are reflected in the treatment goals	181 (84)
	Treatment goals are formulated in concrete terms	215 (86)
Step 5 Planning treatment		113 (52)
	Treatment plan is based on treatment goals	111 (51)

^a^The numbers for the different steps vary due to incomplete recordings

**Table 3:** Patients’ involvement in the PSG

	No	Partly	Yes
*N* = 219	*n* (%)	*n* (%)	*n* (%)
I am aware of my treatment goals	2 (0.9)	21 (9.6)	196 (89.5)
My treatment goals reflect my activity problems	2 (0.9)	20 (9.1)	197 (90)
I am aware how I am going to work on my treatment plan	2 (0.9)	39 (17.8)	178 (81.3)
I feel I have contributed to my treatment plan	3 (1.4)	36 (16.4)	180 (82.2)

As regards to research question 1b: *How satisfied are patients about the PSG?,* the element ‘dose received satisfaction’, i.e. the participants’ satisfaction with the method, was assessed by the question: ‘How satisfied are you about the conversation with the physiotherapist about your problems and treatment?’ Score was on an 11-point scale (0 = not satisfied, 10 = very satisfied).

As regards to research question 1c: *What are the physiotherapists’ experiences with the PSG*?, the element ‘contextual barriers’, i.e. the factors influencing effective implementation of the PSG was assessed by means of a written reflection report (open format) about the physiotherapists’ experiences using the PSG in practice.

Data to explore the impact of the method, for the research question: *‘Did the physiotherapists’ intention to use the PSG, the actual use of the PSG, and their client-centred competences improve?*, were collected prior to the training and during the follow-up, by means of three questionnaires. First, a 31-item questionnaire based on the Theory of Planned Behaviour [28] was used to measure the physiotherapist’s intention to use the PSG. The items were based on our previous analysis of the use of the PSC instrument in practice [6, 8]. It comprised four subscales: attitude (14 items), subjective norms (5 items), perceived behaviour control (9 items), and behaviour intention (3 items), which were scored on a 7-point Likert scale (1 = totally disagree, 7 = totally agree). This questionnaire is available in the online supplement as Additional file 1. Second, the actual use of the PSG was assessed by a 4-item questionnaire on a 11-point scale (0 = not at all, 10 = complete). Questions focussed on the actual use of the ‘new’ elements of the PSG (compared to the original PSC instrument) and concerned the extent to which the physiotherapist had informed the patient about the PSG, had used the PSG to set treatment goals, had involved patients in treatment planning, and had used the PSG to evaluate therapy (lower rows Table 4). Third, the physiotherapist’s client-centred competences were assessed by a 7-item questionnaire that was derived from the ‘Care in Dialogue’ Competency scale [29], using its ‘enabling client participation’ subscale. Items were scored on a 4-point Likert scale (1 = not really, 2 = to a limited extent, 3 = regularly, 4 = very often). This questionnaire is available in the online supplement as supplementary file 2.

**Table 4:** Intention to use the PSG, actual use of the PSG, and client-centred competences, before and after training

	Before	After	Δ	
	*N* = 51	*N* = 47		
Client-centred competences *1–4 Likert scale: 1 = not really, 2 = to a limited extent, 3 = regularly, 4 = very often*	Mean (SD)	Mean (SD)	Mean (SD)	*P* value (CI)^a^
Subscale: enabling client participation (sum score of 7 items, range 7–28)	22.68 (3.09)	23.68 (2.63)	0.66 (1.96)	.026** (.08–1.23)
Intention to use of the PSG *1–7 Likert scale: totally disagree – totally agree*	*N* = 51	*N* = 48		
	Mean (SD)	Mean (SD)	Mean (SD)	*P* value (CI)^a^
Attitude(sum score of 14 items, range: 14–98)	72.85 (9.87)	73.42 (11.69)	0.56 (12.30)	.75 (−3.01–4.13)
Perceived behavioural control(sum score of 9 items, range 9–63)	40.02 (7.19)	41.95 (6.82)	1.94 (7.17)	.07 (−.14–4.02)
	Median (IQR)	Median (IQR)	Median (IQR)	*P* value^b^
Subjective norms(sum score of 5 items, range 5–35)	26.00 (24.00–29.00)	28.00 (25.00–30.00)	1.00 (−1.00–3.00)	.13
Behaviour intention(sum score of 3 items, range 3–21)	15.00 (13.00–18.00)	15.50 (12.00–18.00)	0.50 (−3.00–3.00)	.88
Actual use of the PSG *0–10 scale: 0 = not at all, 10 = complete*	*N* = 51	*N* = 48		
	Median (IQR)	Median (IQR)	Median (IQR)	*P* value^b^
To what extent do you inform the patient about the reason for using the PSG?	6.00 (5.00–7.00)	8.00 (7.00–8.00)	1.00 (0.00–2.00)	.00*
To what extent do you use the PSG to set treatment goals?	8.00 (6.00–9.00)	8.00 (7.00–9.00)	0.00 (0.00–1.00)	.07
To what extent do you involve patients in planning treatment?	7.00 (6.00–8.00)	8.00 (7.00–9.00)	1.00 (0.00–2.00)	.00*
To what extent do you use the PSG to evaluate treatment?	7.00 (6.00–9.00)	8.00 (7.00–8.00)	0.00 (−1.00–1.00)	.25

Δ: Difference: after-before; *CI*: 95% Confidence Interval; *IQR*: Inter Quartile Range: 25–75%; ^a^: paired-samples t-test; ^b^: non-parametric Related Samples Wilcoxon Signed-Rank test; * *P* < 0.01; **P < 0.05

### Data analysis

Quantitative data regarding research questions 1a and 1b were analysed by descriptive statistics. Two independent research assistants were trained and scored all tapes independently. In case of disagreement the first author decided on the final score. The patient files were assessed by a research assistant and all checked by the first author. The tapes and files were used to assess whether and how the steps of the PSG had been performed and recorded [27]. This was scored using standardized observation forms that were based on the learning aims (Table 2). The steps of the PSG method were scored as ‘present’ or ‘absent’. As only an initial consultation was recorded, step 6 of the PSG, i.e., evaluation, was not performed. The patient files were used to score steps 1, 4 and 5.

Qualitative data regarding research question 1c were analysed by conventional content analysis [30]. The physiotherapists’ reflection reports were inductively coded by two researchers (AS, AK). They independently read all reports, marked relevant text fragments that matched the research question about the physiotherapists’ experiences with the PSG, and independently coded these themes. After initial coding, consensus about the coding themes was found by discussion.

Quantitative data regarding research question 2 were analysed using the sum scores for intention to use the PSG, and the client-centred competences. The data collected through the scales were tested for internal consistency using Cronbach’s Alpha (fair: α > 0.7; good: α > 0.8). Data regarding the actual use of the PSG were analysed for each individual question. Data were tested for normality with the Shapiro-Wilk test. Depending on the distribution, the changes in the sum scores were analysed by a paired-samples t-test, or the non-parametric Related Samples Wilcoxon Signed-Rank test. Statistical Software Package for Social Sciences (SPSS) version 23 (SPSS Inc., Chicago IL) was used.

## Results

Data were collected until May 2016. Fifty-one physiotherapists (25 male, 26 female) from 18 different community-based practices participated in the study, mean age: 38 years (SD 12, range: 21 to 60), mean working experience: 14 years (SD: 10, range: 0.5 to 35). They had all been using the original PSC instrument in their daily practice: 16 (31%) used it for about half of their patients and 35 (69%) for more than half of their patients. The participants were trained in four separate groups. Forty-four participants attended all three training sessions, six attended two sessions, and one attended one session. Reasons for absence were illness, holidays and pregnancy.

### Research question 1a: To what extent was the PSG performed as intended?

Observed performance of the PSG could be assessed in 38 recorded consultations (12 video, 26 audio), stemming from 38 physiotherapists. Some of the recordings were incomplete with regard to the presence of the five steps, as the PSG was applied over more than one consultation. Other tapes appeared not to concern an initial consultation, as the physiotherapist was already familiar with the patient. We analysed 25 complete (including five steps), and 13 incomplete consultations, so not all steps of the PSG were equally present.

The research assistants showed agreement regarding their scoring of the steps 1, 2 and 3, but were often not unanimous in their scoring of steps 4 and 5. They made additional comments on the difficult distinction between the ‘setting goals’ and ‘planning treatment’ steps, and on the lack of clarity in the communication about goals and planning within these steps. Table 2 shows that the steps were almost always present, but detailed components of these steps were often absent. This was especially the case for the following components: ‘explaining reason for the relevant step in all steps’; ‘checking the patient’s understanding in steps 4 and 5’; ‘relating physical examination to the problematic activities in step 4’; ‘introducing the “planning treatment” step in advance’; ‘incorporating the patient’s preferences in the treatment plan’ and ‘summarizing the treatment plan’.

Recorded performance of the PSG could be assessed in 218 patient files delivered by 44 physiotherapists. The files included data of 96 men and 113 women (9 missing), mean age: 56 years (SD: 19, range: 14 to 89) The two independent assessors agreed on more than 90% of the files. The results in Table 2 show that, overall, steps 1 and 4 were correctly recorded and that step 5 was only present in half of the files. The treatment goals generally included problem activities, but these were often formulated in terms of a future PSG score, for example: being able to jump during volleyball with a PSG score of 6.

Patients’ involvement in the PSG could be assessed in 219 patient questionnaires delivered by 44 physiotherapists. The majority of the patients (90%) reported that they had felt involved in the PSG in terms of awareness of their treatment goals, and that their activity problems were reflected in these goals. More than 80% knew how they were going to work on the treatment plan and felt that they had contributed to the plan (Table 3).

### Research question 1b: How satisfied are patients about the PSG?

Patients (*n* = 219) rated their satisfaction about the conversation about their problems and treatment at a mean of 8.8 (SD 1.0, range 5 to 10), on a 0–10 scale.

### Research question 1c: What are the physiotherapists’ experiences with the PSG?

The reflection reports regarding the physiotherapists’ experiences with the PSG (*N* = 38) were independently coded by two authors (AS, AK), who reached consensus about four themes: (a) the physiotherapists’ opinion about the PSG itself, (b) their awareness of the importance of patient-centred communication, (c) their perception of the quality of patient participation and (d) their opinion about the impact of the electronic patient system.

As regards (a), all physiotherapists found the steps clear and easy to apply. The integration of the method in the entire physiotherapy process helped them to work in a more structured and goal-oriented way. The reversed scoring was regarded as easier and more suitable for the patients in comparison to the original PSC instrument. The example questions in the instruction manual were regarded as practical. Some physiotherapists perceived the PSG as time-saving, while others felt they did not have enough time to perform the method as intended, and found it difficult to change their routine behaviour.

As regards (b), the physiotherapists felt they had become more aware of the importance of an appropriate use of communication skills, such as specifying things in concrete terms and summarizing them as recommended. They appreciated the process of informing the patient and explaining the reason for the different steps, which was new to them. They also realized that they needed more time to change their behaviour and to improve their communication skills in order to stimulate patients to participate.

As regards (c), the majority of the physiotherapists perceived increased patient participation, in terms of patients being actively involved and enjoying the discussion about their treatment and goals. This resulted in shared decisions about goals and treatment plans. However, they also reflected that some patients preferred not to become involved, but preferred the physiotherapist to take the lead. They also found patient participation was not increased for patients with cognitive or communication problems, because they could not formulate or specify problematic activities in concrete terms.

As regards (d), the main barrier to using the PSG in practice was that it was hard to fit the method in the current electronic patient systems, as it was difficult to record and score treatment goals in the system.

### Research question 2: Did the physiotherapists’ intention to use the PSG, the actual use of the PSG, and their client-centred competences improve?

Table 4 shows the results of the three questionnaires.

#### Intention to use of the PSG

The internal consistency was good for the subscales intention and attitude (α > 0.8), and fair for the subscales subjective norms and perceived behaviour control (α > 0.7). The paired-samples t-test was used to analyse the subscales: attitude and perceived behaviour control, the non-parametric Related Samples Wilcoxon Signed-Rank test was used to analyse the subscales: subjective norms and behaviour intention. The mean and median sum scores on the subscales showed no significant improvement after follow-up.

#### Actual use of the PSG

The non-parametric Related Samples Wilcoxon Signed-Rank test was used. After follow-up, there were small, but significant improvements in ‘informing patients about the reason for using the PSG’ (*P* < 0.01) and ‘involving patients in treatment planning’ (*P* < 0.01).

#### Client-centred competences

The internal consistency of the subscale was fair (α > 0.7). The paired-samples t-test showed a small but significant improvement (*P* < 0.05) in the mean sum score after the training course.

## Discussion

We evaluated the feasibility of a new goal-setting method, the PSG, in physiotherapy. The extent to which the PSG was actually applied was high, as regards to the separate steps, but the ‘setting goals’ and ‘planning treatment’ steps were not performed in detail. Patients were satisfied about the method and felt involved, i.e., they were aware about treatment goals and treatment plan. The physiotherapists’ overall experiences were positive and they regarded the PSG as useful to stimulate patient participation. They became more aware of the importance of communication and engaging patients in a dialogue, and realized that their paternalistic attitude limited shared decision making. A main barrier for effective implementation was that the method did not fit into the current electronic patient systems. Regarding to the second aim of the study, we saw only minor improvements in the self-reported actual use of the ‘new elements’ of the PSG, i.e., on informing and involving patients, and small improvement in the client-centred competences.

The improvement in the self-reported actual use was not completely confirmed by their objectively observed performance in the recorded sessions. This might be explained in two ways. First, the physiotherapists had already been using the original PSC instrument, and there may have been an optimistic bias towards their own performance prior to the training. They scored relatively high, leaving not much room for improvement. After the training, they may then become more critical about their competence and recognized their deficit, resulting in relatively lower improvement of the self-reported scores. The second possibility is that although the physiotherapists were facilitated by their practice managers to participate in the training, they may have been mostly extrinsically motivated by the quality credit points they received for their participation, and we may have overestimated their readiness to change. However, the improved actual use (as regards informing patients and involving them in treatment planning), together with the perceived increased client-centred competences, can be interpreted as signs that the method is being accepted and as a first step in behaviour change.

The discrepancy between the physiotherapists’ perception of patient participation and the actually observed patient participation has also been studied elsewhere [6, 15]. One explanation might be the physiotherapists’ lack of flexibility in responding to patients’ preferences and needs [31]. Physiotherapists might feel uncomfortable, or are not yet used to, sharing responsibilities, due to the focus on their own perspective as an expert and a strong desire to treat [32]. As a consequence they prioritize ‘doing’ over ‘being with’, which is the essence of client-centred care [33]. This might be driven by the dominance of a more biomedical perspective that limits the adoption of client-centred care [31].

Although elements of shared decision making were incorporated in the ‘planning treatment’ step, the physiotherapists did not fully involve the patients in this step, as has also been found in other studies [6, 15, 32, 34]. The physiotherapists reported that not all patients wanted to participate, and this variety as regards patients’ preferences and abilities for participation has also been reported previously [6, 8, 11, 35–37]. On the other hand, we also saw that patients were not always invited to participate, but were nevertheless satisfied about their conversations with the physiotherapists. However, patients are known to be often very satisfied about physiotherapy treatment, regardless of the level of their participation [36].

Although the research assistants were previously trained, the scoring of the presence of the ‘setting goals’ and ‘planning treatment’ steps appeared to be more difficult than scoring the first three steps. This might be explained that the first three steps were more clearly recognizable, because all physiotherapists were familiar with these steps out of the original PSC instrument. The finding that the ‘setting goals’ and ‘planning treatment’ steps were difficult to distinguish, and communication about goals and plan was often non-specific and unclear, indicates the difficulty of performing these steps, as has also been reported in other studies [6, 9, 12, 15, 17, 38]. The missing data from the observation of the video/audiotapes might represent the heavy workload of the physiotherapists for delivering data for the process evaluation, but can also indicate the difficulty of using the PSG as intended.

The less than optimal recording of the treatment plan in the patient files might be due to the structure of the electronic patient files, which lack a separate field for the treatment plan. This made it difficult for us to analyse the patient files, and one might doubt whether it was the recording of the PSG steps we analysed, or the quality of the electronic patient system. Furthermore, the prevailing external audit requirements induce physiotherapists to focus on completing the electronic patient system, and distract from goal setting together with the patient.

### Strength and weaknesses of the study

A major strength of this process evaluation was the systematic planning, designing and execution of the study in accordance with Saunders et al. and Moore et al. [27, 39]. The key elements of process evaluation were operationalized in the first research questions [27]. A weakness of the study might be that we only evaluated the first five steps of the PSG and did not assess the last evaluation step, so we did not gather information about the feasibility of the entire method.

The physiotherapists were purposively sampled from different practices and regions in the country. It was a heterogeneous group which differed in terms of skills and attitude towards the PSG. During the training sessions we observed that some physiotherapists kept thinking in terms of problems instead of opportunities, while others were eager to learn and adopted the new ideas in a natural way. This fair-sized group seems sufficiently representative of physiotherapists working in community-based practices in the Netherlands, given the balance in age, gender and working experience.

Another strength of our study were the multiple methods and sources of qualitative and quantitative data collection we used (methodological and data triangulation) [40]. They enabled us to obtain comprehensive insights into the feasibility and potential impact of the PSG. A limitation is that most of the data were gathered by means of self-assessments, which can cause bias due to social desirability. This was compensated for by the observations, which made it possible to compare the objectively observed performance and the self-reported actual use. To reduce social desirability, the patients were asked to hand their questionnaires to the physiotherapists in a sealed envelope, and all participants were informed about the anonymous analysis of the questionnaires. Although we used several data sources for the physiotherapists, we used only one source for the patients, which was another limitation. Furthermore, patients’ satisfaction was only assessed by one single question, and it might be possible that the meaning of the question a was not clear to the patients. This could mean that patients commented on different aspects of the conversation except the PSG.

The measurement instruments were pretested in a pilot study [24]. To ensure reliability of the findings, the observations and patient files were scored independently, as recommended by Moore et al. [39]. The intended use of the PSG was assessed by the Theory of Planned Behaviour questionnaire [28, 41], with the content of the items being designed on the basis of the previous qualitative analysis [6, 8].

The training was carefully developed. The learning aims were based on the four main objectives for improvement of the PSC instrument, and both trainers were experienced moderators [42, 43]. However, the physiotherapists may not have had enough opportunities to improve sufficiently within this relatively limited follow-up period. Although, the assessments were aimed at the learning aims, these may have been too ambitious to be fully achieved. Extended follow-up and in-service coaching may be needed. Additionally, training and evaluating communication performances is difficult [44], and the importance of devoting attention to good communication is once again confirmed by our findings [45–48]. The fact that some physiotherapists already knew the patient whose session they recorded may also have contributed to our finding that not all steps were fully performed.

We evaluated the improvement in the physiotherapists’ intention to use the PSG, actual use of the PSG, and their client-centred competences by means of a non-controlled intervention study, without a control group or randomization. Therefore the results have to be interpreted as preliminary impressions of the impact of the PSG.

## Conclusion

The PSG is a feasible method for goal setting. Implementation in physiotherapy practice is promising as patients were positive and physiotherapists perceived its benefits. Moreover, physiotherapists realized that good communication skills and changing their routine behaviour, though difficult, are essential to apply the PSG as intended. The method tends to have impact on patient participation, however, its full potential for shared goal setting has not been utilized yet. More effort is needed to achieve the required behaviour change and a truly client-centred attitude, to make physiotherapists totally ready for shared goal setting.

## Implications for practice, research and education

Physiotherapists can already use some elements of the PSG by informing patients, stimulating them to participate and focus on having a dialogue. There may be a need for methods prior to the first consultation, that prepare patients for participation and encourage them to reflect on their reason for consulting and potential treatment goals. The PSG needs to be integrated in the Dutch electronic physiotherapy patient systems. The effectiveness of the PSG should be further studied in clinical trials to see whether has to be investigated. The essential elements of the PSG can be integrated in current Bachelor’s and post-graduate programmes in physiotherapy education, addressing communication skills and adopting a client-centred attitude.

## Additional file

Additional file 1:
**S1** Questionaire: Intention to use the PSG,31-item questionnaire to measure the change in the ‘Intention to use the PSG’. **S2** Questionnaire: Client-centred competences: enabling client participation, 7-item questionnaire to measure the change in the ‘client-centred competences’. (DOCX 23 kb)

## Abbreviations

CI: Confidence Interval
IQR: Inter Quartile Range
KNGF: Koninklijk Nederlands Genootschap Fysiotherapie
PSC: Patient Specific Complaints instrument
PSFS: Patient Specific Functional Scale
PSG: Patient-Specific Goal-setting method
SD: Standard Deviation
SPPS: Statistical Software Package for Social Sciences
