# Supplementary material for: Ready for goal setting? Process evaluation of a patient-specific goal-setting method in physiotherapy
Source: BMC Health Serv Res. 2017 Aug 31;17:618. doi: 10.1186/s12913-017-2557-9 (PMC5579955; doi:10.1186/s12913-017-2557-9)
Supplement: Additional file 1: — S1 Questionaire: Intention to use the PSG,31-item questionnaire to measure the change in the ‘Intention to use the PSG’. S2 Questionnaire: Client-centred competences: enabling client participation, 7-item questionnaire to measure the change in the ‘client-centred competences’. (DOCX 23 kb) [file 12913_2017_2557_MOESM1_ESM.docx]

**Additional files:**

**Supplementary file 1**

Questionaire: Intention to use the PSG

31-item questionnaire to measure the change in the ‘ Intention to use the PSG’.

Supplementary file 2

Questionnaire: Client-centred competences: enabling client participation

7-item questionnaire to measure the change in the ‘ client-centred competences’.

Supplementary material 1

| **Questionnaire: Intention to use the PSG** | |
| --- | --- |
| **Attitude** | |
| 1 | Using the PSG contributes to my client-centred approach |
| 2 | Using the PSG contributes to the patients’ awareness of their own functioning |
| 3 * | Patients do not want to participate in designing their treatment plan |
| 4 | The PSG helps me to set treatment goals |
| 5 * | I think that using the PSG takes too much time |
| 6 | Patients are able to make a shared decision about treatment goals |
| 7 | The PSG helps me to set up the treatment plan |
| 8 | Evaluating with the PSG provides me and the patient with insights into the treatment results |
| 9 | Using the PSG increases the patient’s participation in treatment |
| 10 | Scoring activities supports me in understanding the patients’ perception of their problems |
| 11 | Patients are able to make a shared decision about the treatment plan |
| 12* | Patient have problems mentioning problematic activities |
| 13* | Patients do not want to make a shared decision about treatment goals |
| 14 | In my opinion, the PSG must be used for all (chronic) patients |
| **Subjective norms** | |
| 15 | Patients find it important to participate in therapy |
| 16 | The professional community regard it as important to set treatment goals together with the patient |
| 17 | My colleagues also set treatment goals together with their patients |
| 18 | The professional community regard it as important that physiotherapists design a treatment plan together with the patient |
| 19 | My colleagues also design a treatment plan together with their patients |
| **Perceived behavioural control** | |
| 20 | It’s easy for me to use the PSG |
| 21 | I decide for myself whether to use the PSG or not |
| 22* | I find it difficult to specify activities in concrete terms |
| 23* | Using the PSG does not depend on the patient’s capabilities (e.g. cognitive, communicative) |
| 24 | I find it easy to involve patients in designing a treatment plan |
| 25* | Using the PSG depends on the time I have available |
| 26* | I find it difficult to set concrete treatment goals |
| 27 | I feel confident about using the PSG correctly |
| 2* | I find it difficult to involve patients in setting treatment goals |
| **Behaviour intention** | |
| 29 | I want to use the PSG for all my patients |
| 30 | I intend to use the PSG for all my patients |
| 31 | I expect to use the PSG for all my patients |
| **: Negatively formulated questions; scores are reversed* | |

Supplementary material 2

| **Questionnaire: Client-centred competencies: enabling client participation** [1] | |
| --- | --- |
| 1 | I am convinced that patient’s preferences are the starting point for the physiotherapy treatment |
| 2 | I stimulate patients to feel responsible for their own treatment |
| 3 | I stimulate patients to contribute their own knowledge and experience to the treatment |
| 4 | I stimulate patients to actively direct the treatment and make decisions about it |
| 5 | I stimulate patients to organize aspects of their treatment for themselves |
| 6 | I support and encourage patients in their initiatives regarding treatment |
| 7 | I adjust the treatment plan according the patients’ wishes |

1. Schoot T, Hirschman M., de Witte, L. Development of competencies aimed at client-centred care: an evaluation study. Learning in Health and Social Care. 2007;6(2):1-14.
